# Editorial: Innate Immunity in Kidney Injury, Repair and Fibrosis

**DOI:** 10.3389/fimmu.2022.909654

**Published:** 2022-05-05

**Authors:** Bin Yang, Cheng Yang, Ying Wang

**Affiliations:** ^1^ Department of Cardiovascular Sciences, College of Life Sciences, University of Leicester, University Hospitals of Leicester National Health Service (NHS) Trust, Leicester, United Kingdom; ^2^ Nantong-Leicester Joint Institute of Kidney Science, Department of Nephrology, Affiliated Hospital of Nantong University, Nantong, China; ^3^ Department of Urology, Zhongshan Hospital, Fudan University, Shanghai, China; ^4^ Shanghai Key Laboratory of Organ Transplantation, Shanghai, China; ^5^ Zhangjiang Institute of Fudan University, Shanghai, China; ^6^ Chinese Academy of Sciences (CAS) Key Laboratory of Tissue Microenvironment and Tumor, Shanghai Institute of Nutrition and Health, Chinese Academy of Sciences, Shanghai, China

**Keywords:** fibrosis, inflammation, innate immunity, kidney injury, macrophage, phagocytosis, repair and tubular epithelial cell

Innate immunity, a vital system in animals and humans, is precisely regulated, in which a balance between offensive and defensive actions maintains its homeostasis. The innate immunity is orchestrated by immune cells, cytokines and complement under physiological and pathological conditions. The activity of innate immune cells including dendritic cells (DC), neutrophils, macrophages, and tubular epithelial cells (TEC) and endothelial cells (EC) dominates innate immune responses. These innate immune cells not only participate in the occurrence of injury and inflammation, but also play beneficial roles in post-injury repair. Over-reactive and persistent immune-inflammatory responses result in further injury, mal-repair and fibrosis. On the other hand, the timely clearing damaged cells including apoptotic and necrotic cells, often executed by phagocytes including neutrophils, macrophages and TEC, is beneficial (1). Disclosing the complicated mechanisms of renal injury and repair, and the sophisticated actions of innate immunity will facilitate the strategy of limiting inflammation, promoting repair, and preventing fibrosis in both native and transplant kidneys.

Acute kidney injury (AKI) is a life-threatening disease, which affects 10%-15% hospitalized patients and 50% intensive care patients without effective early diagnosis and cause-specific treatment worldwide. AKI also predisposes to chronic kidney disease (CKD) that unavoidably develops to end stage renal disease, with 2 million patients dying each year worldwide due to a shortage of donor kidneys for transplant (2–4). Leading causes of AKI include ischemia-reperfusion (IR) and increased usage of nephrotoxic drugs, in which TEC and EC are the most vulnerable cells and important innate immune cells that regulate injury, repair and potential fibrosis (5). It is imperative to develop cause-specific interventions that will benefit patients clinically, healthcare organizations financially and socioeconomic structures globally. Macrophages are central in the pathogenesis of kidney diseases, and also have therapeutic potential in limiting tissue injury and preventing fibrosis. The infiltration of macrophages, a major subset of innate immune cells, is a common feature after renal IR injury. The infiltrating macrophages can polarize into two distinct types: classically activated M1 macrophages, which not only inhibit infection, but also accelerate renal injury, while alternatively activated M2 macrophages, a repair phenotype, promote wound healing and subsequent fibrosis Xie et al. TSC1 is a negative regulator of the mammalian target of rapamycin (mTOR) signaling that regulates macrophage polarization in inflammation associated diseases. Hu et al. found that myeloid cell-specific TSC1 knockout mice had more severe functional and histological damage in the kidney than wild type (WT) mice during the early phase of IR injury with upregulated most M1 macrophage-related genes, but showed attenuated renal fibrosis during the repair phase with decreased M2 macrophage markers. These findings were confirmed in hypoxia-reoxygenation *in vitro* model. Therefore, TSC1 in macrophages contributes to the whole process of IR injury, serving as an inflammation suppressor during the early injury phase, but a fibrosis promoter over the repair. Liang et al. also reported that macrophages are main infiltrating cells in the kidney of lupus nephritis. PD-1 ligands (PD-Ls) are beneficial by contributing to M2 macrophage polarization and immunosuppression. Total glucosides of paeony (TGP) significantly decreased urinary protein and improved renal function, reducing serum anti-ds-DNA and ameliorating renal immunopathology in lupus nephritis murine model. TGP increased the frequency of splenic and peritoneal M2-like macrophages. TGP may be a potent drug to treat lupus nephritis by IL-4/STAT6/PD-L2 signaling pathway to induce M2-like macrophages. Liu et al. using C57BLKS/6J ^Lep^ *db/db* mice and a high glucose-induced bone marrow-derived macrophage polarization system showed that hyperoside (HPS), an active flavonoid glycoside in the Chinese herbal medicine Tu-Si-Zi, markedly reduced diabetes-induced albuminuria and glomerular mesangial matrix expansion, with a significant improvement of fasting blood glucose level, hyperlipidaemia and body weight. The pretreatment of HPS effectively regulated macrophage polarization by shifting M1 to M2 phenotype, resulting in the inhibition of M1 macrophage infiltration, reducing pre-inflammatory mediators including MCP-1, TNF-α and iNOS, and increasing anti-inflammatory cytokine Arg-1 and CD163/CD206. HPS also promoting CD4^+^ T cell differentiation into Th2 and Treg populations. Wen et al. reviewed recent studies and highlighted that kidney macrophages are notably heterogeneous immune cells that fulfil opposing functions in terms of clearing deposited pathogens, maintaining immune tolerance, initiating and regulating inflammatory responses, and degrading the extracellular matrix, or promoting kidney fibrosis. Macrophage origins can partially explain macrophage heterogeneity in the kidneys. Circulating Ly6C^+^ monocytes are recruited to inflammatory sites by chemokines, while self-renewed kidney resident macrophages contribute to kidney repair and fibrosis. Mechanisms underlying kidney macrophage heterogeneity may help develop macrophage-targeted therapies for kidney diseases. Saisorn et al. postulated that IR might be exacerbate lupus activity through neutrophil extracellular traps (NETs) and apoptosis. They performed renal IR injury in Fc gamma receptor 2b deficient (Fcgr2b-/-) lupus mice. At 24 h IR injury, NETs in peripheral blood neutrophils and in kidneys, and apoptosis in kidneys were more prominent in Fcgr2b-/- mice compared to WT. After 120 h renal IR injury, renal NETs were non-detectable, whereas glomerular immunoglobulin deposition and serum anti-dsDNA were increased in Fcgr2b-/- mice, implying that raised NETs at the early stage of IR injury have long-term impact on exacerbating lupus nephritis. Moreover, acute or chronic kidney diseases often cause micronutrient deficiency including reduced folate, an essential B vitamin, in which reabsorption of folate by TEC is important. Yang et al. reported that low plasma folate in rats subjected to kidney IR injury, which were inversely correlated to plasma creatinine, with decreased expression of folate transporters. Inhibiting folate transporter in human TEC reduced intracellular folate. These results suggest that IR injury downregulates folate transporters and folate uptake by TEC, and leads to low folate in the circulation.

Innate immunity plays multiple roles in kidney repair. Properdin, a positive regulator of complement alternative pathway, participates in renal IR injury, and also acts as a pattern-recognition molecule facilitating the phagocytic clearance of damaged cells. Wu et al. revealed that properdin knockout (P^KO^) mice exhibited greater injury than WT mice post 72-h IR, with more apoptotic cells and macrophages in tubule luminal areas, and further raised EPOR. Properdin siRNA reduced properdin expression in H_2_O_2_ treated TEC, but raised apoptosis. The phagocytic ability of WT TEC was promoted by H_2_O_2_, but inhibited by P^KO^, while only locally produced properdin plays crucial roles in opsonizing damaged cells and regulating TEC phagocytosis. Moreover, major causes of AKI including IR, not only induce injury, but also initiate self-defense system to limit injury and promote repair. One of these innate immune responses is to produce erythropoietin (EPO) that simultaneously triggers its homodimer receptor (EPOR)_2_ and heterodimer EPOR/β common receptor (EPOR/βcR), also called innate repair receptor (6). EPOR/βcR is expressed in several cell types including TEC at low level, but is swiftly upregulated and also translocated to cellular membrane under stress such as IR injury. EPOR/βcR mediates anti-apoptosis, anti-inflammation, pro-regeneration, and remodeling in AKI *via* different signaling pathways including PI3K/Akt, STAT3 and MAPK. Wu and Yang also reviewed the biological functions and mechanistic signaling pathways of these receptors in AKI, and discuss its potential clinical applications as a biomarker for early diagnosis, predicting prognosis, and guiding cell target drug delivery. AKI requires more efficient and specific treatments, rather than just supportive therapy. Mesenchymal stem cells (MSCs) are promising for cellular therapy because of their low immunogenicity and ability to expand *in vitro*. The main therapeutic effects of MSCs were mediated by MSC-derived extracellular vesicles (EVs) that even have more merits including lower immunogenicity, no tumorigenesis and artificially modifying potential. Li et al. reviewed the therapeutic efficacy and mechanism of MSCs and MSC-EVs in AKI and believe that MSC-EVs will become an effective approach to overcome the current limitations in AKI treatment, although it still face many challenges such as lack of clinical trials so far. Han et al. also reviewed recent studies that have provided a deeper understanding on the mechanisms of drug-induced AKI, among which acute tubular interstitial injury induced by the breakdown of innate immunity plays an important role. MSCs can inhibit kidney damage by regulating innate immunity, promote repair and prevent fibrosis. They addressed the immune pathogenesis of drug-induced AKI versus IR-induced AKI, and explored the immunomodulatory effects and therapeutic potential of MSCs for drug-induced AKI. Luo et al. further reviewed MSCs controlled the imbalance between T helper 17 cell (Th17)-mediated pro-inflammatory response and regulatory T cell (Treg)-mediated anti-inflammatory effect in renal TEC injury. Given that Th17 and Treg are derived from a common CD4^+^ T cell precursor, MSC-mediated inhibition of mTOR to restrain CD4^+^ T cell differentiation into Th17, but in turn promotes Treg generation. Therefore, MSC-mediated Th17-to-Treg polarization acts as a new immunotherapy for kidney injury. Furthermore, B cells, commonly regarded as proinflammatory antibody-producing cells, are detrimental to individuals with autoimmune diseases. However, it has been also revealed that regulatory B (Breg) cells, an immunosuppressive subset, may exert protective effects by producing immune-regulatory cytokines including IL-10, TGF-β, and IL-35. The functions of Breg cells vary according to their phenotype, but no specific marker or Breg cell-specific transcription factor has been identified. Long et al. reviewed the phenotypes and function of Breg cells and highlighted their potential therapeutic value in kidney diseases.

Renal fibrosis is the final common pathway of CKD regardless of etiology, in which innate immunity is crucial. Yin et al. demonstrated that parkinson disease protein 7 (PARK7) protects against unilateral ureteric obstruction (UUO)-induced CKD and renal fibrosis by inducing SOD2 to reduce transforming growth factor-b (TGFB1) associated oxidative stress in tubular cells. Innate immune cells, of course, are key contributors to kidney inflammation and fibrosis. *Cormican and Griffin.* reviewed the interactions of CX3CL1 and CX3CR1 in recruiting innate immune cells into the kidney, which that mediates the progression of CKD. They highlighted the therapeutic potential of targeting CX3CL1 or CX3CR1 to benefit CKD patients. In addition, metabolic syndrome refers to the pathological state of metabolism disorder of protein, fat, carbohydrate and other substances in human body. The kidney is an important organ of metabolism, in which various metabolic disorders can cause inflammatory response and kidney damage. Xiong et al. reviewed the function and specific regulatory mechanism of inflammasomes including NLRP3 in inflammatory infiltration, pyroptotic cell death and kidney damage caused by various metabolic disorders, and provide a new therapeutic perspective and targets for kidney diseases. Li et al. found for the first time that fibroblastic reticular cells (FRCs) increased production of extracellular matrix (ECM) fibers and remodeled the microarchitecture of the UUO kidney-draining lymph node (KLN) in mice, contributing to fibrosis that mirrored the changes in the kidney. The populations of CD11b^+^ antigen-presenting cells, CD11c^+^ DC, and activated CD4^+^ and CD8^+^ T cells were also significantly higher in the UUO KLN than that in the KLN draining the unaffected contralateral kidney, with upregulated TGFβ/TGFβR signaling pathway. Both release of ureteral ligation at 2 days following UUO and depletion of FRCs at the time of injury onset halted the progression of fibrosis in both the kidney and the KLN. Gui et al. found that everolimus (EVR) could attenuate the progression of epithelial-mesenchymal transition (EMT) and renal allograft interstitial fibrosis, and also activate autophagy in chronic allograft dysfunction rat model. EVR may retard impaired autophagic flux and block NF-κB pathway activation, and thereby prevent progression of TNF-α-induced EMT and renal allograft interstitial fibrosis. Zheng et al. reported hydroxychloroquine (HCQ) efficiently inhibited the activation of macrophages and MAPK signaling pathways, and attenuated renal fibrosis in a mouse model of IR-induced renal tubulointerstitial fibrosis. HCQ also inhibited macrophage activation *in vitro*, especially M2 macrophages, and promoted macrophage apoptosis. The effects of HCQ on renal fibrosis and macrophages were decreased after depletion of TLR-9. Therefore, HCQ could be a new anti-fibrotic drug and TLR-9 could be a potential therapeutic target for CKD following AKI. Wu et al. found microRNA (miR)-146a-5p was the most significant up-regulated miRNA in the TEC of renal fibrosis mouse model induced by repeated low-dose cisplatin, while Tfdp2 gene was one target gene of miR-146a-5p. Bone marrow MSCs attenuate cisplatin-induced renal fibrosis by regulating the miR-146a-5p/Tfdp2 axis in mouse renal TEC.

Innate immunity is also important in kidney transplantation. Renal IR injury and cyclosporine A (CsA) nephrotoxicity affect allograft function and survival. Zheng et al. showed that CHBP, only initiating EPOR/βcR, predominantly protects kidneys against IR injury at 2 weeks and/or CsA nephrotoxicity at 8 weeks in mouse kidneys, with different underlying mechanisms. Urinary albumin/creatinine is a good biomarker in monitoring the progression of transplant-related injuries. CsA divergently affects apoptosis in kidneys and cultured kidney epithelial cells, in which CHBP and/or CASP-3 siRNA reduces inflammation and apoptosis. Antibody-mediated rejection (AMR) represents a major cause of allograft dysfunction and results in allograft failure in solid organ transplantation. CHBP ameliorated renal allograft rejection in a renal transplantation model. Zheng et al. investigated the effect of CHBP on AMR using a secondary allogeneic skin transplantation model by transplanting skin from BALB/c mice to C57BL/6 mice. Skin allograft survival was significantly prolonged by CHBP, with reduced CD19^+^ B cells, splenic plasma cells, germinal center B cells, and Tfh cells in skin allografts, and ameliorated serum donor specific antibodies. CHBP inhibited AMR, which may be mediated by NF-*κ*B signaling to suppress B cell immune responses. Acute and chronic AMR are directly mediated by B cells and difficult to treat. Long-lived plasma cells (LLPCs) in bone marrow play a crucial role in the production of the antibodies that induce AMR. LLPCs survive through a T cell-independent mechanism and resist conventional immunosuppressive therapy. Matsuda et al. reviewed the direct or indirect involvement of immunocompetent cells in the differentiation of naïve-B cells into LLPCs, the development and limitations of control methods for AMR, and their potential clinical applications. Wu et al. analyzed publicly available RNA-sequencing datasets from three mouse bulk kidney mRNA-sequencing including IR or cisplatin induced AKI and UUO induced fibrosis, and one human single-cell kidney RNA-sequencing. Regardless of causes of AKI, T cell activation was mainly related to renal fibrosis progression, while fatty acid metabolic process and arachidonic acid metabolism mainly occurred in tubule cells. The differences in the AKI-to-fibrosis process caused by different factors were connected to the different functions of immune cells and tubule cells in the kidney, which may pave the way for exploring novel potential therapeutic strategies. It is well known that the survival of transplant kidneys using deceased donor (DD) kidneys is inferior to living donor (LD) kidneys. Yang et al. conducted a microarray analysis of 24 human DD and LD kidney biopsies paired at 30 minutes and 3 months post-transplantation. 446 differentially expressed genes (DEGs) between DD and LD at 30 minutes reflected donor injury and acute immune responses, while 146 DEGs at 3 months represented adaptive immunity and immunosuppressive treatment. Some early expressed DEGs correlated with renal function and histology at 12 months were further validated by qPCR in additional 33 time point unpaired kidney biopsies. Divergent transcriptomic signatures between DD and LD might contribute to the allograft survival of two type donors. DEGs including SERPINA3 could be novel biomarkers for diagnosis and intervention over donor kidney preservation. The immune rejection is effectively controlled in the acute phase of kidney transplantation, but chronic rejection mediated by chronically activated antibodies ultimately lead to graft failure. At present, immunosuppressive agent fails to increase the long-term survival rate of allografts, and also has many adverse reactions. Zhuang et al. reviewed the latest prospects of immune cells focusing on regulatory myeloid cells to regulate the immune response in renal transplantation, and also introduce their respective progress from experimental research to clinical immunotherapy to control allograft rejection. Lin et al. also reviewed DC induce and regulate adaptive immunity in the kidney by discussing their origin, maturation, pathological effects, and interaction with effector T cells and Tregs. Divergent DCs play both positive and negative roles in IR-induced renal transplant injury, in terms of switching phenotypes to result in acute or chronic rejection, and orchestrating surface markers for allograft tolerance *via* alterations in metabolism. A multidimensional transcriptomic analysis of renal transplantation will facilitate the understanding DC ontogeny and subpopulations.

## Conclusions

On the Research Topic of “Innate Immunity in Kidney Injury, Repair and Fibrosis”, 16 research articles and 11 review papers were published, which addressed the involvement of macrophage polarization, neutrophils, TEC and DC, regulatory myeloid cells and B cells, and kidney-draining lymph node in a broad range of acute and chronic renal injury models induced by IR, UUO, cisplatin, lupus and diabetes, as well as immune response associated DEGs in biological samples from kidney transplant patients. In addition, a variety of relevant therapeutic interventions including erythropoietin derived peptide CHBP, everolimus and Chinese medicine hyperoside in limiting injury, promoting repair and preventing fibrosis were validated in different disease models *via* different mechanisms and signalling pathways associated with innate immunity cells and mediators. We believe this Research Topic provides new findings of innate immunity in both native and transplant kidney diseases. We much appreciate all contributions from authors, reviewers and editors alike.

## Author Contributions

BY, CY and YW wrote the editorial. All authors contributed to the editorial and approved the submitted version.

## Conflict of Interest

The authors declare that the research was conducted in the absence of any commercial or financial relationships that could be construed as a potential conflict of interest.

## Publisher’s Note

All claims expressed in this article are solely those of the authors and do not necessarily represent those of their affiliated organizations, or those of the publisher, the editors and the reviewers. Any product that may be evaluated in this article, or claim that may be made by its manufacturer, is not guaranteed or endorsed by the publisher.
